# Bayesian feature selection for radiomics using reliability metrics

**DOI:** 10.3389/fgene.2023.1112914

**Published:** 2023-03-08

**Authors:** Katherine Shoemaker, Rachel Ger, Laurence E. Court, Hugo Aerts, Marina Vannucci, Christine B. Peterson

**Affiliations:** ^1^ Department of Mathematics and Statistics, University of Houston-Downtown, Houston, TX, United States; ^2^ Department of Radiation Oncology and Molecular Radiation Sciences, Johns Hopkins University School of Medicine, Baltimore, MD, United States; ^3^ Department of Radiation Physics, The University of Texas MD Anderson Cancer Center, Houston, TX, United States; ^4^ Artificial Intelligence in Medicine (AIM) Program, Mass General Brigham, Harvard Medical School, Boston, MA, United States; ^5^ Department of Radiation Oncology, Brigham and Women’s Hospital, Harvard Medical School, Dana-Farber Cancer Institute, Boston, MA, United States; ^6^ Radiology and Nuclear Medicine, CARIM & GROW, Maastricht University, Maastricht, Netherlands; ^7^ Department of Statistics, Rice University, Houston, TX, United States; ^8^ Department of Biostatistics, The University of Texas MD Anderson Cancer Center, Houston, TX, United States

**Keywords:** Bayesian modeling, classification, quantitative imaging, probit prior, radiomics, variable selection

## Abstract

**Introduction:** Imaging of tumors is a standard step in diagnosing cancer and making subsequent treatment decisions. The field of radiomics aims to develop imaging based biomarkers using methods rooted in artificial intelligence applied to medical imaging. However, a challenging aspect of developing predictive models for clinical use is that many quantitative features derived from image data exhibit instability or lack of reproducibility across different imaging systems or image-processing pipelines.

**Methods:** To address this challenge, we propose a Bayesian sparse modeling approach for image classification based on radiomic features, where the inclusion of more reliable features is favored *via* a probit prior formulation.

**Results:** We verify through simulation studies that this approach can improve feature selection and prediction given correct prior information. Finally, we illustrate the method with an application to the classification of head and neck cancer patients by human papillomavirus status, using as our prior information a reliability metric quantifying feature stability across different imaging systems.

## 1 Introduction

Imaging is a key step in the diagnosis, staging, and assessment of treatment response in cancer. Patient images, which may be collected using x-ray, computed tomography (CT), magnetic resonance (MR), or other imaging systems, are typically interpreted by a radiologist. However, relying on humans to review medical images has critical limitations, including time, expense, and variability among image readers. The field of radiomics aims to use quantitative methods to characterize images, essentially considering them as a form of high-dimensional data. A large number of radiomic features can be automatically extracted from the image that can then be used in the development of diagnostic, predictive, or prognostic models.

In this work, we propose a novel approach for the classification of patients based on radiomic features derived from imaging data. Our method relies on Bayesian priors to favor the selection of features that have been shown in previous studies to be more robust to extraneous aspects of image acquisition and processing. To lay the groundwork for our proposed method, we begin with a review of radiomics and relevant statistical modeling approaches in Section 2. In Section 3, we introduce our proposed sparse classification model, which can predict a patient’s group membership based on radiomic features. To improve estimation accuracy and interpretability of the model, we rely on a Bayesian variable selection framework to identify features that are relevant to the classification task, favoring the inclusion of features that are more robust to extraneous sources of variation. Specifically, we use a probit prior to incorporate information on feature stability. In Section 3.5, we describe posterior inference and prediction. In Section 4.1, we compare the performance of our proposed approach to alternative methods in terms of variable selection and classification accuracy. We conclude with a case study in Section 4.2 illustrating the application of our model to classify head-and-neck cancer patients based on radiomic data, identifying quantitative imaging features that differ by HPV status.

## 2 Background

### 2.1 Radiomics

**FIGURE 1:**
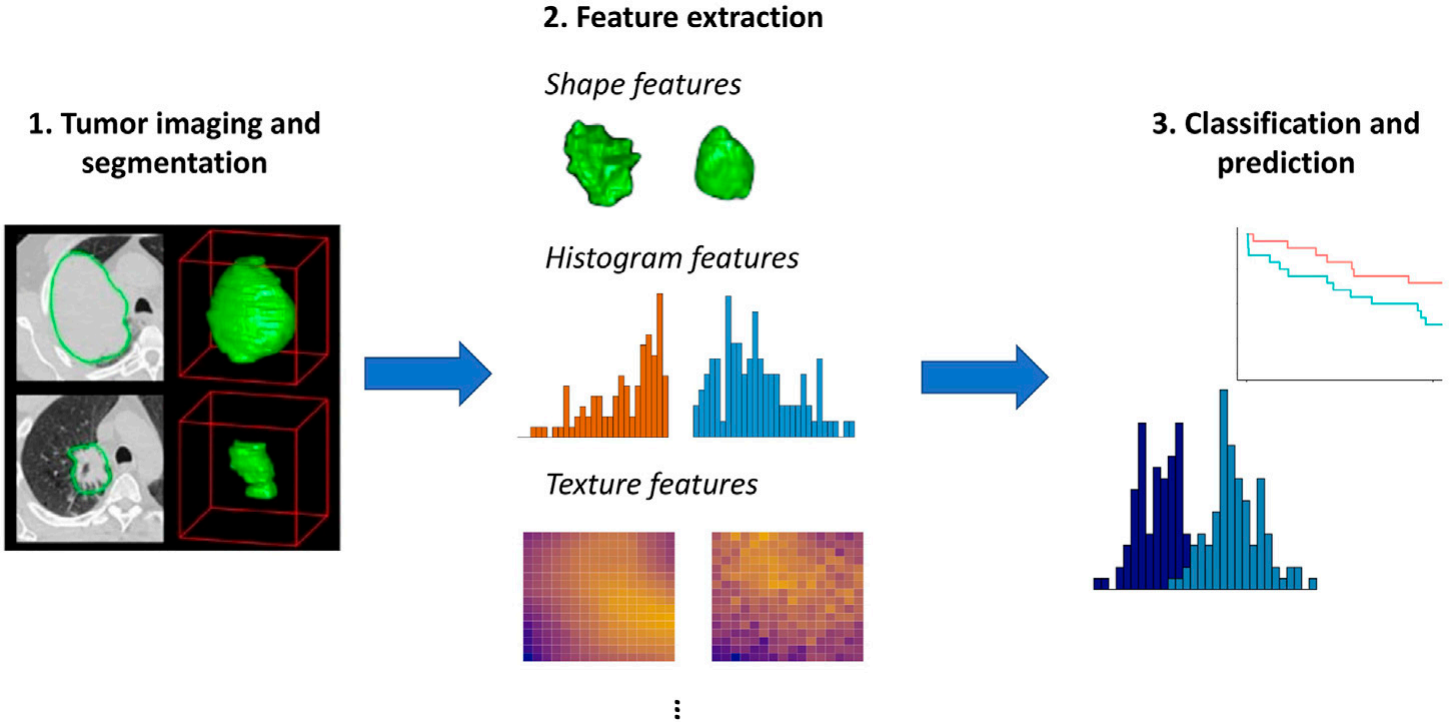
Overview of radiomics. Here, we use images from Aerts et al. (2014) to illustrate the initial step of tumor imaging and segmentation. Quantitative features can then be extracted that summarize the tumor boundary (shape features), the distribution of the pixel intensity values within the tumor (histogram features), and aspects of the spatial relations among pixels with differing intensities (texture features). More advanced features such as model- or tree-based summaries may also be computed. These features can then be used as input to approaches aimed at prediction of survival outcomes or classification methods such as mixture models, as illustrated generically in Step 3.

Radiomics is a framework for medical image analysis that entails extracting large numbers of quantitative features from imaging data (Lambin et al., 2012; Gillies et al., 2016). As illustrated in Figure 1, these features can then be used to objectively characterize aspects of the tumor, group patients with similar imaging features, and predict outcomes such as survival or response to treatment. It has been hypothesized that radiomic features derived from imaging data may reflect molecular and genomic characteristics of a patient’s tumor (Aerts et al., 2014). The idea that advanced analytics on images can capture important information on a patient’s tumor biology and prognosis is called the radiomics hypothesis (Lambin et al., 2012).

There are many factors motivating the development of the field of radiomics. A standard approach to ascertain molecular features of the tumor is to biopsy specific locations within the tumor; this approach is not only invasive, but may fail to capture the heterogeneity of the tumor beyond the sites assayed. In particular, it has been noted that quantifications of molecular features such as protein expression exhibit spatial and regional differences when multiple biopsies are taken within a single tumor (Van Meter et al., 2006). To get a more comprehensive view, radiomic features summarizing the entire tumor in a medical image can be extracted (Aerts et al., 2014). These radiomic features are objectively assessed and can be used to develop models for diagnosis, classification, or prediction. Although not currently in wide use clinically, radiomics is gaining traction in the clinical sphere, with great interest in developing diagnostic tools and personalized medicine approaches (Lambin et al., 2017).

As illustrated in Figure 1, a first step in the analysis is segmentation of the tumor, or delineation of the tumor boundaries. Various radiomics features can then be extracted using tools such as IBEX (Zhang et al., 2015) or PyRadiomics (Van Griethuysen et al., 2017):

• *Shape features*, including volume, surface area, sphericity, and compactness, summarize morphological aspects of the tumor. Unsurprisingly, volume tends to be a useful predictor; however, its performance as a biomarker can be improved by considering additional radiomic features (Aerts et al., 2014).
• *Histogram features*, also known as first order features, summarize the distribution of pixel intensity values, without consideration of their position. Histogram features include standard univariate summary measures such as mean, median, minimum, maximum, standard deviation, skewness, and entropy.
• *Texture features* summarize spatial relations among pixels. These features describe the tendency of pixels with similar intensities to occur nearby (gray level co-occurrence) and the number of pixels in a row or the size of regions with shared intensities (gray level run length and gray level size zone).
• *Advanced features*, including model-based Mayerhoefer et al. (2020) and tree-based features Shoemaker et al. (2018) have also been proposed.

Current work on predictive modeling from radiomics features relies heavily on machine learning methods such as support vector machines and random forests (Hawkins et al., 2014; Parmar et al., 2015; Vallières et al., 2017). Although models derived from radiomics data show promising performance, they have not yet filtered into clinical practice, as it is difficult for clinicians to understand these models and feel confident in the results. In their critical review paper, Morin et al. (2018) argue that in order to realize the potential for radiomics models to be used clinically, researchers must focus on clarity and interpretability, moving away from black-box methods towards more transparent modeling approaches. In recent years, deep learning approaches for image segmentation and predictive modeling have shown incredible promise, but the black-box nature of deep learning methods remains a hurdle to the acceptance of their use in clinical decision making (Rogers et al., 2020).

In addition to the need for interpretable models, another concern for clinical translation of radiomics models is that many radiomics features are dependent on aspects of the image acquisition and processing (Scalco and Rizzo, 2016). Essentially, differences in radiomics feature values may not only arise from aspects of the tumors being imaged, but also from extraneous aspects of the image collection and processing. This can make it challenging to validate radiomics models across institutions. Previous work has highlighted various sources contributing to this instability. In particular, it has been shown that radiomic features are influenced to varying extents by differences in the imaging system used (Ger et al., 2018) as well as by processing steps including the image reconstruction algorithm (Zhao et al., 2016) and the choice of how to quantize the image data (Desseriot et al., 2017). Traverso et al. (2018) provide an overview of research on repeatability and reproducibility of radiomic features, including both phantom and human studies for a variety of cancer types. Subsequent work has explored the use of image perturbations to quantify radiomic feature robustness (Zwanenburg et al., 2019). In an important first step towards reproducibility, the Image Biomarker Standardization Initiative (IBSI) has recently developed standardized names, definitions, and reference values for a core set of radiomic features, but differences in image acquisition and processing prior to radiomic feature calculation remain challenges (Gillies et al., 2016; Morin et al., 2018; Zwanenburg et al., 2020). Critically, these differences can result in instability and a lack of reproducibility. While many studies on predictive modeling using radiomics features ignore this issue, some authors have chosen to filter out features with low reproducibility prior to model building (Velazquez et al., 2017). Rather than screening features upfront, in the current work, we propose a more flexible approach to model building that can account for feature reliability as a continuous value.

### 2.2 Bayesian variable selection

In high-dimensional data applications, variable selection methods can be applied to encourage sparse solutions and enable the identification of a “best” subset of predictors. By reducing dimensionality and filtering out potentially irrelevant features, sparse modeling approaches can improve predictive accuracy, mitigate issues with collinearity, and allow for the interpretation of the model through the investigation of selected features (Hastie et al., 2009). In the frequentist framework, variable selection may be achieved through the use of penalties on model parameters, as in the lasso (Tibshirani, 1996) or elastic net (Zou and Hastie, 2005). In the Bayesian framework, variable selection entails the choice of appropriate priors on the model parameters (Tadesse and Vannucci, 2021). Broadly speaking, Bayesian variable selection approaches offer a number of attractive qualities as compared to alternative statistical and machine learning methods, including the ability to quantify uncertainty regarding model and feature selection, the ability to incorporate prior information in the model construction, and the fact that parameters can be automatically chosen through the use of hyperpriors, avoiding the need for cross-validation.

We now discuss prior work on Bayesian variable selection in more detail. Bayesian variable selection approaches include mixture priors (George and McCulloch, 1993, 1995), Bayesian analogs to the lasso (Park and Casella, 2008) and elastic net (Li and Lin, 2010), and global-local shrinkage priors such as the horseshoe (Carvalho et al., 2009), horseshoe+ (Bhadra et al., 2017), and regularized horseshoe (Piironen and Vehtari, 2017). In the mixture prior framework, the inclusion of each variable in the model is directly represented *via* a latent indicator. Stochastic search algorithms (George and McCulloch, 1995) can then be applied to identify models with high posterior probability. In addition to the regression setting (George and McCulloch, 1993), mixture priors have been incorporated for feature selection in clustering and classification problems (Tadesse et al., 2005; Stingo et al., 2013). Shrinkage priors offer some improved computational scalability, since they do not require sampling of latent indicator variables; when using shrinkage priors, feature selection can be achieved based on criteria such as whether posterior credible intervals overlap zero, and prior information (for example, on the expected degree of model sparsity) can be incorporated through the choice of hyperparameters. In the mixture model framework, the explicit representation of variable inclusion allows for the formulation of informative priors on the latent indicator variables and posterior model selection *via* thresholding of posterior probabilities of feature inclusion. In low-dimensional settings, the maximum *a posteriori* (MAP) model may be considered as the “best” model. For high-dimensional settings, where the space of potential models is quite large, it makes more sense to focus on the marginal posterior probabilities of inclusion for each feature. The median probability model, which includes all features with marginal posterior probability greater than 0.5, is a popular choice, as it has been shown to be optimal for prediction in settings with Gaussian noise (Barbieri and Berger, 2004).

As mentioned above, the ability to incorporate prior knowledge in feature selection is a key advantage of the Bayesian framework. In the mixture prior setting, the inclusion of feature *j* in the model can be represented using a latent binary variable *γ*_*j*_. To reflect a preference for sparsity, the prior probability of variable inclusion *π*(*γ*_*j*_ = 1) can be assumed to follow a Bernoulli distribution with a small mean, such as 0.05. Including an additional layer in the hierarchical prior specification by placing a Beta prior on the Bernoulli parameters has been shown to provide automatic adjustment for multiplicity (Scott and Berger, 2010). Alternatively, if information on individual features or their interrelation is available, this can be reflected in a more tailored prior specification. In early work in this area, Chipman (1996) described the formulation of Bayesian priors for models with interaction terms, grouped predictors, and competing predictors. Li and Lin (2010) and Stingo et al. (2011) encourage the joint selection of predictors that are related within a network using a Markov random field prior. Finally, Quintana and Conti (2013) propose a hierarchical probit model that allows for the integration of multiple sources of information on the model covariates.

Here, we propose a sparse Bayesian model for image classification based on radiomic features. We refer to this method as RVS, for radiomic variable selection. Elements of the model including the mixture formulation with selection of discriminatory features build on Tadesse et al. (2005) and Stingo et al. (2013). However, there are key differences of the current model from prior work. In particular, Stingo et al. (2013) proposed a hierarchical model with selection of upstream factors influencing the discriminatory features, while we focus on the integration of external information on feature reliability *via* the probit prior.

## 3 Methods

### 3.1 Classification model

In this section, we describe the structure of the observed data and the formulation of the model, including the likelihood and priors. Let **X** represent the *n* × *p* matrix of radiomics data, where *j* = 1, … , *p* indexes the radiomic feature, and *i* = 1, … , *n* indexes the subject. We also observe the *n*-vector of class membership ***g***, where *g*_*i*_ ∈ {1, … , *K*}. This class membership may correspond to disease subtype or any other categorization of the subjects into *K* groups.

We assume that only a subset of the *p* features are relevant to the classification problem. By assuming a sparse model, we reduce noise in prediction and are able to identify a set of important variables. We use the latent binary vector ***γ*** = (*γ*_1_, … , *γ*_*p*_) to represent the feature selection. Specifically, *γ*_*j*_ = 1 indicates that the *j*th feature is useful in discriminating the subjects into groups, while *γ*_*j*_ = 0 indicates the *j*th feature is not relevant to the classification problem. This leads to the mixture model:

$$f_{k}\left(x_{ij}|\gamma_{j}\right)=\left(1-\gamma_{j}\right)f_{0}\left(x_{ij};\theta_{0j}\right)+\gamma_{j}f\left(x_{ij};\theta_{kj}\right),$$ (1)

where the term *f*_0_(*x*_*ij*_; *θ*_0*j*_) represents the distribution of the non-discriminatory “noise” features, and *f*(*x*_*ij*_; *θ*_*kj*_) represents the distribution in group *k* of the differential features relevant to the classification task, for *k* = 1, … , *K*. Here, *θ*_0*j*_ represents the parameters of the distribution of a non-discriminatory feature, while *θ*_*kj*_ represents the group-specific parameters of the distribution of a differential feature.

Assuming that the radiomic features have been transformed to improve normality if appropriate and centered at 0, we take the distributions *f*_0_ and *f* to be the following Gaussian densities: $f_{0}\left(x_{ij};\theta_{0j}\right)=N\left(0,\sigma_{0j}^{2}\right)$ and $f\left(x_{ij};\theta_{kj}\right)=N\left(\mu_{kj},\sigma_{kj}^{2}\right)$ . Based on the latent indicator vector ***γ***, the matrix **X** can be split into **X**_(*γ*)_, composed of the features such that *γ*_*j*_ = 1, and $X_{\left(\gamma^{c}\right)}$ , containing the features for which *γ*_*j*_ = 0. Using the model in Eq. 1, it follows that ***x***_*i*(*γ*)_ follows a multivariate normal distribution conditional on the group assignment *g*_*i*_, and $x_{i\left(\gamma^{c}\right)}$ follows a multivariate normal distribution which is not conditional on the group assignment. If we let *p*_*γ*_ represent the total number of selected features, we can write:

$$\begin{array}{ll} x_{i\left(\gamma \right)}|g_{i}=k & ∼N\left(\mu_{k\left(\gamma \right)},\Sigma_{k\left(\gamma \right)}\right)\\ x_{i\left(\gamma^{c}\right)} & ∼N\left(0,\Omega_{0\left(\gamma^{c}\right)}\right), \end{array}$$ (2)

where **Σ**_*k*(*γ*)_ and $\Omega_{0\left(\gamma^{c}\right)}$ are diagonal matrices, specifically, **Σ**_*k*(*γ*)_ = $\text{Diag}\left(\sigma_{k1}^{2},\ldots ,\sigma_{kp_{\gamma }}^{2}\right)$ and $\Omega_{0\left(\gamma^{c}\right)}$ = $\text{Diag}\left(\sigma_{01}^{2},\ldots ,\sigma_{0\left(p-p_{\gamma }\right)}^{2}\right)$ . For simplicity of notation, we assume that the variables are rearranged such that the *p*_*γ*_ selected variables are followed by the (*p* − *p*_*γ*_) non-selected variables. In this formulation, the discriminatory features are allowed to have both a group-specific mean and a group-specific variance, while the non-discriminatory features are assumed to come from a distribution that is not group-specific, and therefore share a common mean of zero after centering and a common variance. Relaxing the assumption that **Σ**_*k*(*γ*)_ is diagonal could allow additional flexibility to model the correlations among the discriminatory features within each class *k*; these could potentially be linked across classes through a hierarchical prior. For the variance terms in Eq. 2, we place the following inverse-gamma priors on the diagonal elements: $\sigma_{kj}^{2}\;|\;a_{k},b_{k}∼IG\left(a_{k},b_{k}\right)$ , and $\sigma_{0j}^{2}\;|\;a_{0},b_{0}∼IG\left(a_{0},b_{0}\right)$ .

### 3.2 Variable selection using a probit prior

The latent indicator vector ***γ*** represents the selection of features for use in the classification problem. As mentioned in Section 2.1, radiomic features are often characterized by high variation across imaging systems or parameters in image reconstruction and processing. To develop models with robust predictive performance across settings, we would like to favor the inclusion of predictors which are the most reliable, ones that vary the least from machine to machine. Specifically, we place a probit prior that can take into account the stability of each feature as quantified by previous computational or phantom studies. For *j* = 1, … , *p*, we place an independent prior on *γ*_*j*_ such that

$$p\left(\gamma_{j}=1\;|\;\alpha_{0},\alpha_{1},N_{j}\right)=\Phi \left(\alpha_{0}+\alpha_{1}N_{j}\right),$$ (3)

where ***N*** is a vector of length *p* that denotes the reliability of each feature. The vector ***N*** = (*N*_1_, … , *N*_*p*_) represents external information on each covariate. Although it is not constrained mathematically, for ease of interpretation, it may make sense to scale the entries of ***N*** to the interval [0,1] such that *N*_*j*_ = 0 reflects a lack of prior preference for feature *j* and higher values reflect stronger prior preference. The parameter *α*_0_ establishes the prior probability of variable inclusion in the case that the reliability metric for that particular feature has the value 0. Specifically, if *N*_*j*_ = 0 for feature *j*, then the prior probability of inclusion for that feature, i.e., for *γ*_*j*_ to be non-zero, reduces to *p*(*γ*_*j*_ = 1 | *α*_0_). We assume that *α*_0_ is a fixed hyperparameter. The parameter *α*_1_ influences the impact of the prior information on the selection. We allow *α*_1_ to be fixed, but if more flexibility is needed, *α*_1_ could be allowed to follow a hyperprior such as $\alpha_{1}∼N\left(w,\tau^{2}\right)$ .

### 3.3 Prior for group-specific means

We now describe the prior distributions for the mean parameter introduced in Eq. 2 above. For the selected variables, we allow the group-specific mean parameters to come from a normal prior:

$$\mu_{k\left(\gamma \right)}∼N\left(\nu_{k\left(\gamma \right)},h_{1}\Gamma_{k\left(\gamma \right)}\right).$$ (4)

To complete the hierarchical prior specification for the group-specific means, we place a normal prior on the mean term and an inverse-Wishart prior on the variance term from Eq. 4: $\nu_{k\left(\gamma \right)}∼N\left(m_{k\left(\gamma \right)},h_{1}\Gamma_{k\left(\gamma \right)}\right)$ and **Γ**_*k*(*γ*)_ ∼IW(*d*_*k*_, **Q**). We assume the scale matrix **Q** is the diagonal matrix *c* ×**I**. The non-selected features have a common prior mean of 0.

### 3.4 Model overview

We now summarize the proposed RVS model, including the likelihood and priors. The following mixture describes the likelihood of the observed radiomic features **X** given group memberships ***g***, and variable selection indicators ***γ***:

$$L\left(X|g,\gamma ,\cdot \;\right)=\overset{K}{\underset{k=1}{\prod }}\underset{i:g_{i}=k}{\prod }\overset{p}{\underset{j=1}{\prod }}\left\{\gamma_{j}N\left(X_{ij};\mu_{kj},\sigma_{kj}^{2}\right)+\left(1-\gamma_{j}\right)N\left(X_{ij};0,\sigma_{0j}^{2}\right)\right\}.$$

**FIGURE 2:**
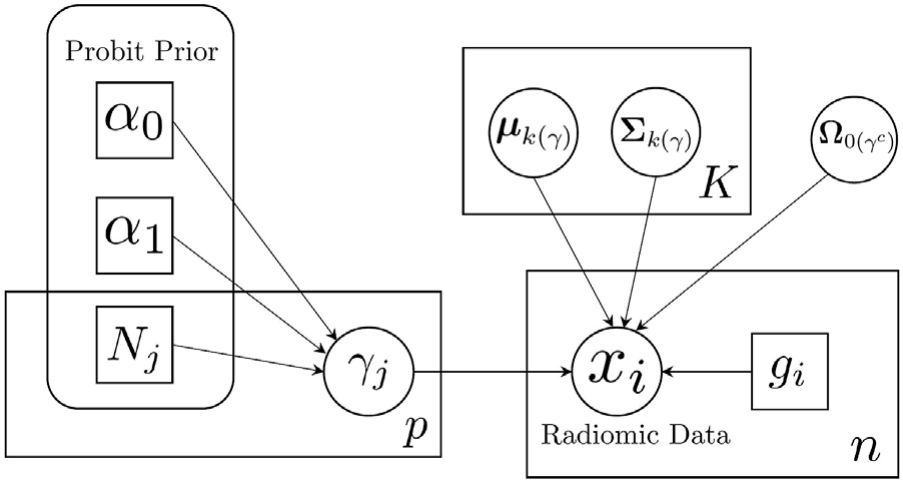
Schematic illustration of sparse Bayesian classification model. Feature-specific prior information is incorporated through the probit prior.

We illustrate our proposed model using a plate diagram in Figure 2, and summarize the full hierarchical model, including the likelihood and priors, in Table 1.

**TABLE 1:** A summary of the RVS model specification.

Likelihood
$L\left(X|g,\gamma ,\cdot \right)=\prod_{k=1}^{K}\prod_{i:g_{i}=k}\left\{N\left(x_{i\left(\gamma \right)};\mu_{k\left(\gamma \right)},\Sigma_{k\left(\gamma \right)}\right)\times N\left(x_{i\left(\gamma^{c}\right)};0,\Omega_{0\left(\gamma^{c}\right)}\right)\right\}$
$\Sigma_{k\left(\gamma \right)}=Diag\left(\sigma_{k1}^{2},\ldots ,\sigma_{kp_{\gamma }}^{2}\right)$
$\Omega_{0\left(\gamma^{c}\right)}=Diag\left(\sigma_{01}^{2},\ldots ,\sigma_{0\left(p-p_{\gamma }\right)}^{2}\right)$
Probit prior on variable selection indicators
$P\left(\gamma \;|\;\alpha_{0},\alpha_{1},N\right)=\prod_{j=1}^{p}\Phi {\left(\alpha_{0}+\alpha_{1}N_{j}\right)}^{\gamma_{j}}\times {\left(1-\Phi \left(\alpha_{0}+\alpha_{1}N_{j}\right)\right)}^{1-\gamma_{j}}$
Priors for selected variable parameters
$\mu_{k\left(\gamma \right)}\;|\;\nu_{k\left(\gamma \right)},\Gamma_{k\left(\gamma \right)}∼N\left(\nu_{k\left(\gamma \right)},h_{1}\Gamma_{k\left(\gamma \right)}\right)$
$\nu_{k\left(\gamma \right)}\;|\;m_{k\left(\gamma \right)},\Gamma_{k\left(\gamma \right)}∼N\left(m_{k\left(\gamma \right)},h_{1}\Gamma_{k\left(\gamma \right)}\right)$
**Γ**_*k*(*γ*)_ | *d*_*k*_, **Q** ∼IW(*d*_*k*_, **Q**)
$\sigma_{kj}^{2}\;|\;a_{k},b_{k}∼IG\left(a_{k},b_{k}\right)$
Priors for non-selected variable parameters
$\sigma_{0j}^{2}\;|\;a_{0},b_{0}∼IG\left(a_{0},b_{0}\right)$

### 3.5 Posterior inference

Since the posterior distribution of the parameters is intractable, we rely on Markov chain Monte Carlo (MCMC) sampling to perform posterior inference. As in Stingo et al. (2013), to simplify the posterior sampling and speed up the computation, parameters including the variances *σ* ^2^ and hyperparameters in Eq. 4 are integrated out. The selection of the discriminatory features (through the variable ***γ***) is then the main objective of the sampling algorithm. MCMC sampling for Bayesian variable selection, generally referred to as stochastic search variable selection, involves searching over the space of likely configurations of the latent indicator variables, and has been successfully applied in a variety of high-dimensional applications (George and McCulloch, 1993; Tadesse et al., 2005). Details on the MCMC algorithm, including the full conditional distributions, sampling steps, and formula for posterior prediction, are provided in Supplementary Section S1.

## 4 Results

### 4.1 Simulation studies

In this section, we describe our exploration of the model’s performance on simulated data sets. The simulation set-up was designed to assess the impact of adding the novel prior on feature selection and classification accuracy, as compared to both a neutral prior setting and alternative frequentist methods. Since the case study described in Section 4.2 entails imbalanced data, in Section 4.1.3, we include a set of simulation studies with unequal group sizes.

#### 4.1.1 Data generation

For each simulated data set, we generated observations corresponding to subjects from two classes with equal sample sizes (*n*_1_ = *n*_2_ = 50) with 4 discriminatory features, generated with $\sigma_{ii}^{t}=1$ and $\sigma_{ij}^{t}=0.1$ , *i* ≠ *j*, and 100 noise features, generated with $\sigma_{ii}^{n}=0.7$ and $\sigma_{ij}^{n}=0.3,i\neq j$ . The means of the discriminatory features were set to −1 and 1 for class 1 and 2, respectively.

#### 4.1.2 Comparison of methods

For both the proposed method and the model with a neutral prior setting, three MCMC chains were run, each with 100,000 iterations and a burn-in of 20,000 iterations. For the proposed method, the hyperprior values for the probit prior were set to *α*_0_ = −2.75 and *α*_1_ = 3. As prior information, we used *N*_*j*_ = 0.35 for the reliability metric of the discriminatory variables, and 0.15 for all other entries in ***N***. For the neutral prior setting, *N*_*j*_ was set to be 0.15 for all variables. With the given setting of *α*_0_ and *α*_1_, these values of *N*_*j*_ correspond to a prior probability of inclusion of 4% when *N*_*j*_ = 0.35 and 1%, when *N*_*j*_ = 0.15. The remainder of the hyperparameter values were set as in Stingo et al. (2013). Specifically, the parameter settings were as follows: *a* = 3, *b* = 0.1, *a*_*k*_ = 3, *b*_*k*_ = 0.1, *c* = 0.5, **Q** = *c*∗**I**_*p*_, *d*_*k*_ = 3, and *h*_1_ = 1. For a discussion of parameter sensitivity, see Section 4.1.4 below. In the MCMC algorithm, the probability of add/delete vs. swap was set to 50/50. For the Bayesian methods, we consider a feature to be selected if its marginal posterior probability of inclusion exceeds 0.5, as this has been shown to be optimal in terms of predictive accuracy (Barbieri and Berger, 2004).

To provide a benchmark for comparison, we also applied lasso logistic regression (Friedman et al., 2010), a frequentist method that relies on penalization to achieve model sparsity, and a support vector machine (SVM), a machine learning model designed for classification tasks that does not perform feature selection. The results of our proposed RVS method, the Bayesian method with a neutral prior, and the lasso in terms of feature selection are provided in Table 2. The results shown are averaged over 100 simulated data sets.

**TABLE 2:** Feature selection: The true positive rate (TPR), false positive rate (FPR), and Matthew’s Correlation Coefficient (MCC) for feature selection, for the balanced simulated data. All metrics are averaged over 100 simulated data sets. The highest MCC value is indicated in bold.

	TPR	FPR	MCC
RVS	0.93	0.0005	**0.95**
Neutral	0.55	0.0004	0.71
Lasso	0.995	0.11	0.52

To assess the performance of these methods in terms of feature selection, we computed the average true positive rate (TPR), false positive rate (FPR), and Matthew’s Correlation Coefficient (MCC), which is a measure of the overall feature selection accuracy that ranges from −1 to 1. The MCC is an informative metric particularly when there are significant size differences between the positive and negative classes, as we have here with 4 discriminatory variables and 100 noise variables (Chicco and Jurman, 2020). We also considered the model’s performance with respect to classification, computing the average TPR, FPR and Youden’s Index for categorizing the observations based on their true class membership. Youden’s Index is a summary measure of classification accuracy frequently used in conjunction with ROC analysis that is computed by sensitivity + specificity − 1, and thus ranges from 0 to 1 (Youden, 1950).

**FIGURE 3:**
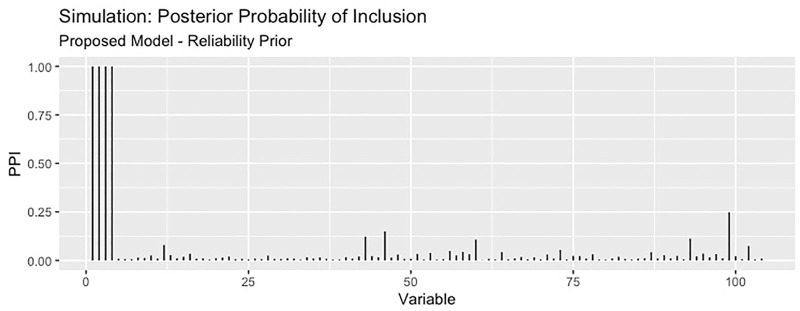
The posterior probability of inclusion for all variables from the proposed method, for one simulated data set. The first four variables are the discriminatory variables, and the remaining 100 are the noise variables.

The proposed RVS method and lasso logistic regression both achieved a high TPR for feature selection. For this simulation setting, both Bayesian methods achieved specificity close to 1 (with average FPR values of 0.0005 and 0.0004, respectively), while the lasso had a higher rate of 0.11. Since there are far more noise variables than discriminatory features in the ground truth, the proposed method achieves the highest MCC for feature selection. An illustration of the marginal posterior probabilities of feature inclusion obtained using RVS for one simulated data set (Figure 3) shows a clear separation between the posterior probabilities of the discriminatory variables (the first four features, with posterior probabilities close to 1) and the remaining noise variables. In this simulation setting, the “neutral” prior setting expressed a strong preference for sparsity without a preference for any individual features; this resulted in a lower TPR and MCC.

**FIGURE 4:**
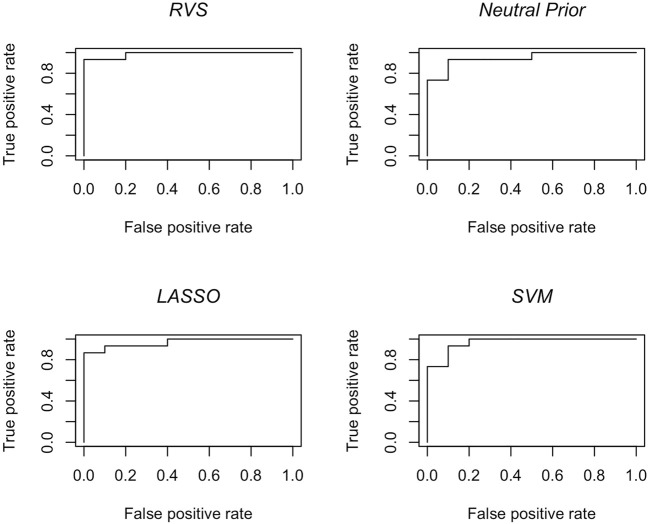
Classification accuracy: ROC curves for the RVS model, the Bayesian model with a neutral prior, lasso logistic regression, and SVM on an example simulated test set, as described in Section 4.1.2.

The results in terms of classification accuracy on simulated test data are provided in Table 3. We provide the mean value for each metric, over the 100 simulated datasets. The proposed RVS method has an improved TPR and FPR over the lasso and SVM, achieving the highest Youden’s index. The four models’ ROC curves for a randomly selected simulation are presented in Figure 4. It is worth noting that the ROC curves show small differences. Indeed, the average AUC value across the 100 simulated datasets was 0.986 for RVS, 0.962 for the Bayesian method with the neutral prior, 0.981 for lasso logistic regression, and 0.970 for SVM. It has been previously noted in the literature that the lasso tends to be insufficiently sparse when using prediction accuracy as the criterion for penalty parameter selection (Leng et al., 2006). This suggests that more advanced methods for tuning the penalty parameter selection could improve the performance of lasso logistic regression (Chen and Chen, 2012).

**TABLE 3:** Classification accuracy: The true positive rate (TPR), false positive rate (FPR), and Youden’s Index for group classification, for the balanced simulated data. All metrics are averaged over 100 simulated data sets. The highest Youden's index value is indicated in bold.

	TPR	FPR	Youden
RVS	0.93	0.06	**0.87**
Neutral	0.89	0.11	0.78
Lasso	0.92	0.08	0.84
SVM	0.90	0.09	0.80

#### 4.1.3 Additional simulation scenarios

**FIGURE 5:**
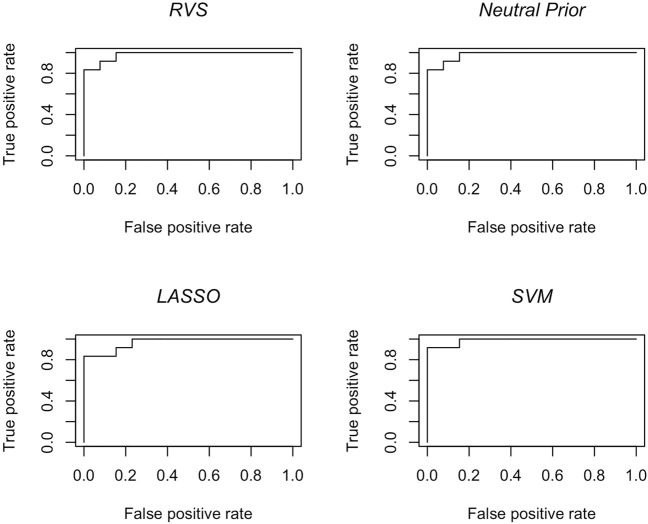
Classification accuracy: ROC curves for the RVS model, the Bayesian model with a neutral prior, lasso logistic regression, and SVM on a test set with unequal group sizes, as described in Section 4.1.3.

To enable performance comparison in a broader range of settings, we constructed additional simulation scenarios. We first considered a simulation on data generated with unequal class sizes: *n*_1_ = 60 and *n*_2_ = 40. The results in terms of feature selection are given in Table 4. The results in Table 5 show that the informative prior allows the proposed RVS method to achieve better classification accuracy than the Bayesian model with the neutral prior, the frequentist lasso method, or SVM. The ROC curves for the four models on a randomly selected simulation dataset can be found in Figure 5. The average AUC value across the 100 simulated datasets was 0.991 for RVS, 0.980 for the neutral prior, 0.980 for the lasso, and 0.969 for SVM.

**TABLE 4:** Feature selection: The true positive rate (TPR), false positive rate (FPR), and Matthew’s Correlation Coefficient (MCC) for feature selection, for the simulated data with unequal group sizes. All metrics are averaged over 100 simulated data sets. The highest MCC value is indicated in bold.

	TPR	FPR	MCC
RVS	0.96	0.001	**0.96**
Neutral	0.72	0.001	0.82
Lasso	0.98	0.10	0.51

**TABLE 5:** Classification accuracy: The true positive rate (TPR), false positive rate (FPR), and Youden’s index for group classification, for the simulated data with unequal group sizes. All metrics are averaged over 100 simulated datasets. The highest Youden's index value is indicated in bold.

	TPR	FPR	Youden
RVS	0.95	0.06	**0.89**
Neutral	0.93	0.08	0.85
Lasso	0.90	0.07	0.83
SVM	0.87	0.08	0.79

To characterize the performance of the methods under a larger variety of scenarios, we performed simulation studies with a stronger class imbalance (90% vs. 10% split), varying number of predictors, varying signal strength, and a varying ratio of discriminatory to non-discriminatory covariates. Across all methods compared, the settings with stronger class imbalance and fewer discriminatory features were more challenging, resulting in lower classification accuracy on the test set. Relative performance was consistent with the simulation study of Section 4.1.2, in that the proposed RVS method achieved the highest MCC for feature selection and highest Youden’s index for classification. Results are provided in Supplementary Section S2.

#### 4.1.4 Sensitivity analysis

Here we provide an overview of parameter sensitivity, performed on the main simulation. Additional details and plots are provided in Supplementary Section S3. Sensitivity analysis was done on three key hyperparameters: *c*, *α*_0_ and *α*_1_. The parameter *c*, the value of the diagonal entries of the scale matrix **Q**, influences the inverse-Wishart hyperprior on the variances of the class-specific means of the features. For values of *c* smaller than 0.3, the model selected a large number of variables. As we varied *c* from 0.3 to 0.7, we found that the FPR and the accuracy only suffered at the larger end of the range, and were fairly consistent otherwise. This is illustrated in the left panel of Supplementary Figure S2, available in Supplementary Section S3.

The parameters *α*_0_ and *α*_1_ modify the impact of the reliability measure for the probit prior on the likelihood of variable selection, *γ*, *p*(*γ*) = Φ(*α*_0_ + *α*_1_∗*N*). In our simulation model, *α*_0_ = −2.75 and *α*_1_ = 3. The plots in Supplementary Figure S2, illustrate the changes in the mean posterior probability of inclusion for the real and noise variables, as we vary the values of *c*, *α*_0_, and *α*_1_. These are generally stable across the range of values considered.

#### 4.1.5 Convergence

To assess convergence of the MCMC chains, we provide traceplots and $\hat{R}$ values for the ***μ*** parameters in Supplementary Section S4. In general, the traceplots suggest good mixing and consistent behavior across chains. We estimated the $\hat{R}$ values using the rhat() function from the posterior R package (Bürkner et al., 2022), which provides the maximum of the rank normalized split- $\hat{R}$ (Gelman et al., 2013) and the rank-normalized folded-split- $\hat{R}$ (Vehtari et al., 2021). The resulting $\hat{R}$ values had a maximum of 1.048 for the simulation study summarized in Section 4.1.2 above.

### 4.2 Case study

We now illustrate the application of the proposed method to a real-world data set aimed at characterizing imaging features associated with head and neck cancer.

#### 4.2.1 Radiomic features of head and neck cancer

There are more than 800,000 new cases of head and neck cancer diagnosed worldwide every year (Cramer et al., 2019). The majority of these cancers are driven by known risk factors including tobacco use, alcohol, and human papillomavirus (HPV) infection (Rettig and D’Souza, 2015; Vokes et al., 2015). Although smoking-related cancers have declined in the US in recent years, as fewer Americans smoke, the incidence of HPV-associated head and neck cancer has increased rapidly during this time. There is a great interest in understanding how radiomic features relate to tumor characteristics and patient prognosis in head and neck cancer. Previous research has linked radiomic features to genomic aspects of the tumor and survival (Aerts et al., 2014; Wang et al., 2020). Recent work (Zhu et al., 2019) on head and neck cancer radiomics has shown that radiomic features can be used to predict HPV infection as well as *TP53* mutation status, which suggests that radiomic features can serve as relevant biomarkers for genomic alterations in the tumor.

In this case study, we consider a data set with radiomic features extracted from CT scans of 102 patients with head and neck cancer. We have clinical information including HPV status, survival time, and staging. It should be noted that smoking information is not available for this cohort. There are 160 radiomic variables, computed in the same manner as those used in the reliability study by Ger et al. (2018), which we combined with two clinical variables, Age and Shape Volume. Our case study investigates the relationship between the radiomic features, the reliability of those features, and the HPV status of the patient. In particular, we applied our proposed Bayesian model as well as the lasso to predict HPV status from the radiomic data. Of the 102 patients, 84 were HPV negative and 18 were HPV positive.

#### 4.2.2 Data processing

The radiomic features were computed using IBEX, an open source radiomics tool (Zhang et al., 2015). As described in Fave et al. (2017), the features were each calculated using four different image preprocessing techniques. It has been shown that the utility of a feature in downstream modeling may depend on preprocessing, but that none of the preprocessing methods are superior in general (Fave et al., 2016). We therefore included features produced using all 4 preprocessing approaches as candidates in our modeling. Since some features were highly skewed, Box-Cox transformations were applied as appropriate to improve normality. Finally, the features were centered and scaled, resulting in distributions that were approximately standard normal.

#### 4.2.3 Prior information

In Ger et al. (2018), the authors scanned a phantom on 100 CT machines produced by various manufacturers in 35 clinics throughout the Texas Medical Center (Ger et al., 2018). Radiomic features were calculated on the 100 scans, and a linear mixed effect model was used to partition the variability due to the manufacturer and to the individual scanners. In this study, we used the standard deviation of the features from the individual scanners as a measure of feature reliability, with the motivation that features that are highly dependent on the individual scanner being used may contribute less relevant information regarding tumor biology.

To use the standard error measure given in Ger et al. (2018) as a reliability metric, we used the following formula to transform the information on the *j*th feature *r*_*j*_:

$$N_{j}=|\log\left(r_{j}\right)-\underset{k}{\max}\left(\log\left(r_{k}\right)\right)|$$

By using this formula, we were able to transform the measure into a reliability metric where higher values correspond to more reliable features. The two clinical values were given the mean value of the measure as their metric. To standardize the values, we scaled the metric from 0 to 1, and this value was used as the value *N*_*j*_ in our probit prior.

#### 4.2.4 Application of the proposed model

The data were randomly split 75%/25% into training and test data sets, resulting in groups of size *n*_train_ = 76 and *n*_test_ = 26. The split was balanced with respect to the classification; the training group was 18% HPV positive, while the test group was 16% HPV positive.

For the proposed Bayesian method, three chains were run for 100,000 iterations with a burn-in period of 20,000 iterations each. As in the simulation study, variable inclusion was determined by thresholding the PPI at 0.5. For both RVS and the Bayesian method with the neutral prior, an additional MCMC chain was run to resample ***μ*** with ***γ*** fixed, to obtain a sample of the mean parameters conditional on the set of selected features. Parameters for the probit prior were set to *α*_0_ = −2.75, and *α*_1_ = 1, to express a strong preference for sparsity; the remainder of the parameters were set as in the simulation study. The reliability vector *N* was set as described above in Section 4.2.3, using the processed values of the standard error of the features between various machines.

As in the simulation study, to provide a comparison for our proposed model, we applied two additional methods. For the neutral prior, we set the prior parameter *N*_*j*_ to the median value of the reliability values, *N*_*j*_ = the median of *N* = 0.39. We also applied lasso logistic regression, with tuning parameter selection *via* the one standard deviation method on 10 fold cross-validation on the training data.

#### 4.2.5 Convergence

We computed $\hat{R}$ values as described in Section 4.1.5 above. The maximum value for RVS across all selected features in both groups was 1.001. The full set of $\hat{R}$ values and corresponding traceplots are provided in Supplementary Section S4.

#### 4.2.6 Results

In terms of feature selection, our proposed model selected 11 of the 162 features (7%). This is a sparse subset, allowing for interpretation of the specific features selected. Of the 11 features, 9 were texture features, and the remaining 2 were histogram features. The 9 texture features included 3 gray-level co-occurrence matrix features, 4 gray-level run-length matrix features, and 2 neighborhood intensity difference features (busyness and coarseness). Texture features have been identified as relevant to prediction of survival in prior studies (Aerts et al., 2014). In particular, high tumor busyness (which reflects rapid changes in intensity between neighboring voxels) has been linked to risk of recurrence (Ahn et al., 2019). Recent work published in *JAMA Oncology* proposed a radiomic signature for survival prediction that included features characterizing spatial heterogeneity and texture. This signature held up to validation across multiple medical centers, suggesting that texture features are clinically relevant and potentially generalizable across different settings (Farwell and Mankoff, 2022).

In terms of accuracy on the test set, the proposed model correctly classified 18 of the 26 test observations, with a sensitivity of 66.6% (4 of 6), specificity of 70% (14 of 20), an overall accuracy of %, and a Youden’s Index of 0.37. Although the Youden’s index is fairly low, it is higher than that obtained from the Bayesian model with the neutral prior or from lasso logistic regression: the model obtained using the neutral prior was even more sparse, with 6 of the 162 features selected, but was less accurate, correctly classifying 15 of the 26 test observations, with a sensitivity of 66.6% (4 of 6), specificity of 55% (11 of 20), an overall accuracy of 57.69%, and a Youden’s Index of 0.22.

For the lasso logistic regression, while the model produced results with 76.9% accuracy overall (20 of the 26), it had a sensitivity of 0% and a specificity of 100%, predicting all observations into the more common class, HPV negative. This result has a Youden’s index of 0. The lasso selected 5 variables, none of which were selected by the informative prior. 3 variables were selected by both the informative and neutral model settings. More details about the prediction accuracy can be found in Table 6. The poor performance of the lasso is likely due to class imbalance in the training data; various methods have been proposed to address the challenge of machine learning on imbalanced data, including oversampling of the minority class, downsampling of the majority class, and more complex schemes that combine these strategies (Chawla et al., 2002).

**TABLE 6:** Confusion matrices for the classification performance of the proposed RVS model, the Bayesian model with a neutral prior, and lasso on the case study data.

		True class		
		Negative	Positive	Total
Prediction	Negative	14	2	16
	Positive	6	4	10
	Total	20	6	26

		True Class		
		Negative	Positive	Total
Prediction	Negative	11	2	13
	Positive	9	4	13
	Total	20	6	26

		True Class		
		Negative	Positive	Total
Prediction	Negative	20	6	26
	Positive	0	0	0
	Total	20	6	26

## 5 Discussion

In the current work, we have proposed a novel approach for incorporating prior information on feature reliability into a Bayesian classification model. The development of this model was motivated by the challenges of radiomic feature data, which may include features that are susceptible to sources of variation related to image processing or scanner type, rather than underlying signal. We have illustrated this method through both the application to simulated and real data. The case study results reflect a split of the available data into training and testing. We expect the benefit of using the proposed method would be even greater when attempting to train a method on data collected at a particular site or institution and apply this model to external data, where systematic differences in scanner type or machine settings could come into play.

The RVS framework is based on a normal mixture model, which assumes that the features are reasonably normally distributed within each class. In practice, radiomic features may exhibit skewness: in this case, a log or Box-Cox transformation may be applied to achieve approximate normality. We adopted this approach as a preprocessing step of our real data application (Section 4.2.2). Extending the RVS model to allow for heavy-tailed or binary features would be of interest in future work.

The proposed method is implemented in Matlab using MCMC methods. In recent years, alternative computational approaches such as variational inference have gained increasing popularity. Variational inference is attractive as it allows model estimation to be framed as an optimization rather than a sampling problem; however, previous work has shown that it may underestimate posterior variance (Blei et al., 2017). Investigation of its properties in our proposed setting would be of interest in future work. Another alternative computational approach would be to implement the model using a probabilistic programming language such as Stan (Gelman et al., 2015). Since Stan does not directly support sampling of discrete parameters, this would require marginalizing out the latent feature selection indicators ***γ***. We already integrate out parameters including $\sigma_{0j}^{2}$ and $\sigma_{kj}^{2}$ to speed up the MCMC sampling; marginalizing over ***γ*** could further improve the efficiency of posterior inference.

## Data Availability

Code implementing the proposed method and radiomic feature values for the case study are available on Github at https://github.com/kshoemaker/RVS.
