# Supplementary material for: Bayesian feature selection for radiomics using reliability metrics
Source: Front Genet. 2023 Mar 8;14:1112914. doi: 10.3389/fgene.2023.1112914 (PMC10030957; doi:10.3389/fgene.2023.1112914)
Supplement: Supplementary file 1 [file DataSheet1.pdf]

Supplementary Material

for “Bayesian feature selection for radiomics using reliability metrics”

Katherine Shoemaker, Rachel Ger, Laurence E. Court, Hugo Aerts, Marina Vannucci, and Christine B. Peterson

1 Posterior inference 1
 1.1 Derivation of the full conditionals 1
 1.2 MCMC algorithm 4
 1.3 Prediction 6
2 Additional simulation scenarios 6
 2.1 Stronger class imbalance 7
 2.2 Varying number of predictors 7
 2.3 Varying signal strength 7
 2.4 Varying ratio of discriminatory to non-discriminatory features 8
 2.5 Timing comparison 9
3 Sensitivity analysis 9
4 Convergence 9

1 POSTERIOR INFERENCE

1.1 Derivation of the full conditionals

In this section, we show the derivations of the full conditionals. Sampling from these equations is what allows us to carry out the MCMC steps summarized in Section 1.2 below. As the data matrix is split into selected and non-selected features, we derive the full conditionals for the non-selected variables $\mathbf{x}_{j(\gamma^c)}$, given the latent binary vector of selection indicators $\boldsymbol{\gamma}$, and for the selected features $\mathbf{x}_{jk(\gamma)}$, given $\boldsymbol{\gamma}$ and the group specific means μ_{jk}, separately. Note that the dimensions group-specific mean vectors $\boldsymbol{\mu}_{k(\gamma)}$ change depending on the number of non-zero elements of $\boldsymbol{\gamma}$. To handle the changing dimension without the use of reversible jump Markov chain Monte Carlo, we place a normal pseudoprior (Carlin and Chib, 1995; Dellaportas et al., 2002) on the prior means for the non-selected variables:

$$\boldsymbol{\mu}_{0(\gamma^c)} \sim \mathcal{N}(\mathbf{0}, \boldsymbol{\Gamma}_{0(\gamma^c)}). \tag{S1}$$

We define the matrix $\boldsymbol{\Gamma}_{0(\gamma^c)}$ as a diagonal matrix with elements $\boldsymbol{\Gamma}_{0(\gamma^c)} = \text{Diag}(\omega^2_{p_\gamma+1}, \ldots, \omega^2_p)$. We assume an inverse-gamma prior on the diagonal terms $\omega_j^2 \sim \text{IG}(\tilde{a}_j, \tilde{b}_j),\ j = p_\gamma + 1, \ldots, p.$

The full conditionals of $\mathbf{x}_{j(\gamma^c)} | \, \boldsymbol{\gamma}$ are computed as follows:

$$\prod_{j:\gamma_j=0}\{\mathbb{P}(\mathbf{x}_{j\gamma^c},\sigma_{0j}^2|\boldsymbol{\gamma})\} = \prod_{j:\gamma_j=0}\left\{\prod_{i=1}^{n}\left[\frac{1}{\sqrt{2\pi}}\exp\left(-\frac{x_{ij}^2}{2\sigma_{0j}^2}(\sigma_{0j}^2)^{-1/2}\right)\right]\frac{\beta_0^{a_0}}{\Gamma(a_0)}(\sigma_{0j}^2)^{(-\alpha_0+1)}\exp\left(-\frac{b_0}{\sigma_{0j}^2}\right)\right\}$$

$$= \prod_{j:\gamma_j=0}\left\{\left(\frac{1}{\sqrt{2\pi}}\right)^{n}\exp\left(-\frac{\mathbf{x}_j^T\mathbf{x}_j}{2\sigma_{0j}^2}(\sigma_{0j}^2)^{-n/2}\right)\frac{\beta_0^{a_0}}{\Gamma(a_0)}(\sigma_{0j}^2)^{(-\alpha_0+1)}\exp\left(-\frac{b_0}{\sigma_{0j}^2}\right)\right\}$$

$$= \prod_{j:\gamma_j=0}\left\{\left(\frac{1}{\sqrt{2\pi}}\right)^{n}\exp\left(-\frac{1}{\sigma_{0j}^2}(\frac{1}{2}\mathbf{x}_j^T\mathbf{x}_j+b_0)\right)\frac{\beta_0^{a_0}}{\Gamma(a_0)}(\sigma_{0j}^2)^{(-\alpha_0+\frac{n}{2}+1)}\right\}$$

As σ_{0j}^2 follows an inverse gamma distribution, we can integrate σ_{0j}^2 out to obtain:

$$\prod_{j:\gamma_j=0}\left\{\left(\frac{1}{\sqrt{2\pi}}\right)^{n}\frac{\Gamma(\alpha_0+\frac{n}{2})}{\Gamma(\alpha_0)}\frac{b_0^{a_0}}{(b_0+\frac{1}{2}\mathbf{x}_j^T\mathbf{x}_j)^{(a_0+\frac{n}{2})}}\right\}.$$

The full conditionals of $\mathbf{x}_{jk(\gamma)}\,|\mu_{jk},\boldsymbol{\gamma}$ are computed as follows:

$$\mathbb{P}(\mathbf{x}_{jk(\gamma)},\sigma_{kj}^2\mid\mu_{0jk},\boldsymbol{\gamma}) = \prod_{i=1}^{n_k}\left[\frac{1}{\sqrt{2\pi}}\exp\left(-\frac{(x_{ijk}-\mu_{jk})^2}{2\sigma_{kj}^2}\right)(\sigma_{kj}^2)^{-\frac{1}{2}}\right]\frac{b_k^{a_k}}{\Gamma(a_k)}(\sigma_{kj}^2)^{-(a_k+1)}\exp\left(-\frac{b_k}{\sigma_{kj}^2}\right)$$

$$= \left(\frac{1}{\sqrt{2\pi}}\right)^{n_k}\exp\left(-\frac{1}{\sigma_{kj}^2}\left[\frac{1}{2}(\mathbf{x}_{kj}-\mathbb{I}_{n_k}\mu_{jk})^T(\mathbf{x}_{kj}-\mathbb{I}_{n_k}\mu_{jk})\right]\right)\times$$

$$(\sigma_{kj}^2)^{-\frac{n_k}{2}}\frac{b_k^{a_k}}{\Gamma(a_k)}(\sigma_{kj}^2)^{(a_k+1)}\exp\left(-\frac{b_k}{\sigma_{kj}^2}\right)$$

$$= \left(\frac{1}{\sqrt{2\pi}}\right)^{n_k}\exp\left(-\frac{1}{\sigma_{kj}^2}\left[b_k+\frac{1}{2}(\mathbf{x}_{kj}-\mathbb{I}_{n_k}\mu_{jk})^T(\mathbf{x}_{kj}-\mathbb{I}_{n_k}\mu_{jk})\right]\right)\times$$

$$(\sigma_{kj}^2)^{-(a_k\frac{n_k}{2}+1)}\frac{b_k^{a_k}}{\Gamma(a_k)}$$

As σ_{kj}^2 follows an inverse gamma distribution, we can integrate σ_{kj}^2 out to obtain:

$$= \left(\frac{1}{\sqrt{2\pi}}\right)^{n_k}\frac{\Gamma(a_k+\frac{n_k}{2})}{\Gamma(a_k)}\frac{b_k^{a_k}}{\left(b_k+\frac{1}{2}(\mathbf{x}_{kj}-\mathbb{I}_{n_k}\mu_{0jk})^T(\mathbf{x}_{kj}-\mathbb{I}_{n_k}\mu_{jk})\right)^{(a_k+\frac{n_k}{2})}}.$$

The full conditional of $\boldsymbol{\mu}_{k(\gamma)}\mid\boldsymbol{\gamma}$ is computed as follows:

$$\mathbb{P}(\boldsymbol{\mu}_{k(\gamma)}, \boldsymbol{\nu}_{k(\gamma)}, \Gamma_{0k(\gamma)} \mid \boldsymbol{\gamma}) = \mathbb{P}(\boldsymbol{\mu}_{k(\gamma)} \mid \boldsymbol{\nu}_{k(\gamma)}, \Gamma_{k(\gamma)})\mathbb{P}(\boldsymbol{\nu}_{k(\gamma)} \mid \boldsymbol{\mu}_{k(\gamma)}, \Gamma_{k(\gamma)})\mathbb{P}(\Gamma_{0k(\gamma)} \mid d_k, \mathbf{Q})$$

Because the model specification states that $\boldsymbol{\mu}_{0k(\gamma)}$ follows a normal distribution with mean $\boldsymbol{\nu}_{k(\gamma)}$ and variance $h_1\Gamma_{k(\gamma)}$, and that $\boldsymbol{\nu}_{k(\gamma)}$ follows a normal distribution with mean $\mathbf{m}_{k(\gamma)}$ and variance $h_1\Gamma_{k(\gamma)}$, we can conclude that

$$\begin{aligned} \boldsymbol{\mu}_{k(\gamma)} - \boldsymbol{\nu}_{k(\gamma)} &\sim = \mathcal{N}(0, h_1\boldsymbol{\Gamma}_{k(\gamma)}) \\ \boldsymbol{\mu}_{k(\gamma)} - \boldsymbol{\nu}_{k(\gamma)} + \boldsymbol{\nu}_{k(\gamma)} &\sim = \mathcal{N}(\boldsymbol{m}_{k(\gamma)}, 2h_1\boldsymbol{\Gamma}_{k(\gamma)}) \end{aligned}$$

So it follows that

$$\begin{aligned} &\mathbb{P}(\boldsymbol{\mu}_{k(\gamma)} \mid \boldsymbol{m}_{k(\gamma)}, \Gamma_{k(\gamma)}) \times \mathbb{P}(\Gamma_{k(\gamma)} \mid d_k, \mathbf{Q}) \\ &= \left(\frac{1}{\sqrt{2\pi}}\right)^{p_\gamma} |2h_1\Gamma_{k(\gamma)}|^{-1/2} \exp\left(-\frac{1}{2}(\mu_{k(\gamma)} - \boldsymbol{m}_{k(\gamma)})^T(\mu_{0k(\gamma)} - \boldsymbol{m}_{k(\gamma)})\right) \times \\ &\quad \frac{|Q|^{\frac{d_k}{2}}}{2^{d_k p_\gamma/2}\Gamma_{p_\gamma}(\frac{d_k}{2})}|\Gamma_{k(\gamma)}|^{(d_k+p_\gamma+1)/2} \exp\left(-\frac{1}{2}\mathrm{tr}(\mathbf{Q}\Gamma^{-1}_{k(\gamma)})\right) \\ &= \pi^{-\frac{p_\gamma}{2}}(2h_1)^{-\frac{p_\gamma}{2}} \frac{|\mathbf{Q}|^{\frac{d_k}{2}}}{2^{(d_k+1)p_\gamma/2}\Gamma_{p_\gamma}(\frac{d_k}{2})}|\Gamma_{k(\gamma)}|^{(d_k+p_\gamma+1+1)/2} \times \\ &\quad \exp\left(-\frac{1}{2}\mathrm{tr}\left(\left[(\boldsymbol{\mu}_{k(\gamma)} - \boldsymbol{m}_{k(\gamma)})(\boldsymbol{\mu}_{k(\gamma)} - \boldsymbol{m}_{k(\gamma)})^T\frac{1}{2h_1} + \mathbf{Q}\right]\Gamma^{-1}_{k(\gamma)}\right)\right) \\ &= \pi^{-\frac{p_\gamma}{2}}(2h_1)^{-\frac{p_\gamma}{2}} \frac{|\mathbf{Q}|^{\frac{d_k}{2}}}{\Gamma_{p_\gamma}(\frac{d_k}{2})} \frac{\Gamma_{p_\gamma}(\frac{d_k+1}{2})}{|\mathbf{Q} + \frac{1}{2h_1}(\mu_{k(\gamma)} - \boldsymbol{m}_{k(\gamma)})(\mu_{k(\gamma)} - \boldsymbol{m}_{k(\gamma)})^T|^{\frac{d_k+1}{2}}} \end{aligned}$$

And finally, we derive the full conditional for $\boldsymbol{\mu}_{0(\gamma^c)} \mid \boldsymbol{\gamma}$:

$$\begin{aligned} &\mathbb{P}(\boldsymbol{\mu}_{0(\gamma^c)} \mid \boldsymbol{\Gamma}_{0k(\gamma^c)}) \times \mathbb{P}(\boldsymbol{\Gamma}_{0k(\gamma^c)} \mid \tilde{a}, \tilde{b}) \\ &= \prod_{j=p_\gamma+1}^{p} \left[\frac{1}{\sqrt{2\pi}}(\omega_j^2)^{-1/2} \exp\left(-\frac{\mu_{0jk}^2}{2\omega_j^2}\right) \frac{\tilde{b}_j^{\tilde{a}_j}}{\Gamma(\tilde{a}_j)}(\omega_j^2)^{-(\tilde{a}_j+1)} \exp\left(-\frac{\tilde{b}_j}{\omega_j^2}\right)\right] \end{aligned}$$

$$= (\frac{1}{\sqrt{2\pi}})^{p-p_\gamma} \prod_{j=p_\gamma+1}^{p} \left[(\omega_j^2)^{-(\tilde{a}_j+1/2+1} \exp\left(-\frac{1}{\omega_j^2}\left(\frac{\mu_{0jk}^2}{2}+\tilde{b}_j\right)\right) \frac{\tilde{b}_j^{\tilde{a}_j}}{\boldsymbol{\Gamma}(\tilde{a}_j)} \right]$$

$$= \left(\frac{1}{\sqrt{2\pi}}\right)^{p-p_\gamma} \prod_{j=p_\gamma+1}^{p} \frac{\tilde{b}_j^{\tilde{a}_j}}{\left(\frac{\mu_{0jk}^2}{2}+\tilde{b}_j\right)^{(\tilde{a}_j+\frac{1}{2})}} \frac{\boldsymbol{\Gamma}(\tilde{a}_j+\frac{1}{2})}{\boldsymbol{\Gamma}(\tilde{a}_j)}$$

These derived equations are used in the MCMC algorithm to sample from the posterior distribution, as described in the next section.

1.2 MCMC algorithm

Here we provide the joint marginal posterior distribution of the parameters $(\boldsymbol{\gamma},\ \boldsymbol{\mu}_{0k})$ where $\boldsymbol{\mu}_{0k} = (\boldsymbol{\mu}_{k(\gamma)}, \boldsymbol{\mu}_{0(\gamma^c)})$. The unnormalized full conditional distributions of the model, which were derived in Section 1.1 above, are summarized below:

$$\mathbb{P}(\boldsymbol{\gamma}, \boldsymbol{\mu}_{0k} \mid \mathbf{X}) \propto \prod_{k=1}^{K} \prod_{j:\gamma_j=1} \mathbb{P}(\mathbf{x}_{jk(\gamma)} \mid \mu_{jk}, \boldsymbol{\gamma}) \prod_{j:\gamma_j=0} \mathbb{P}(\mathbf{x}_{j(\gamma^c)} \mid \boldsymbol{\gamma}) \times \prod_{k=1}^{K} \mathbb{P}(\boldsymbol{\mu}_{k(\gamma)} \mid \boldsymbol{\gamma}) \prod_{j:\gamma_j=0} \mathbb{P}(\mu_{j(\gamma^c)} \mid \boldsymbol{\gamma}) \tag{S2}$$

with

$$\mathbb{P}(\mathbf{x}_{jk(\gamma)} \mid \mu_{jk}, \boldsymbol{\gamma}) = \left(\frac{1}{\sqrt{2\pi}}\right)^{n_k} \frac{\Gamma(a_k+\frac{n_k}{2})}{\Gamma(a_k)} \frac{b_k^{a_k}}{\left(b_k+\frac{1}{2}(\mathbf{x}_{jk}-\mathbf{1}_{n_k}\mu_{jk})^T(\mathbf{x}_{jk}-\mathbf{1}_{n_k}\mu_{jk})\right)^{(a_k+\frac{n_k}{2})}},$$

$$\mathbb{P}(\mathbf{x}_{j(\gamma^c)} \mid \boldsymbol{\gamma}) = \left(\frac{1}{\sqrt{2\pi}}\right)^{n} \frac{\Gamma(\alpha_0+\frac{n}{2})}{\Gamma(a_0)} \frac{b_0^{a_0}}{\left(b_0+\mathbf{x}_j^T\mathbf{x}_j\right)^{(a_0+\frac{n}{2})}},$$

$$\mathbb{P}(\boldsymbol{\mu}_{k(\gamma)} \mid \boldsymbol{\gamma}) = \pi^{-\frac{p_\gamma}{2}}(2h_1)^{-\frac{p_\gamma}{2}} \frac{|\mathbf{Q}|^{\frac{d_k}{2}}}{\Gamma_{p_\gamma}(\frac{d_k}{2})} \frac{\Gamma_{p_\gamma}(\frac{d_k+1}{2})}{|\mathbf{Q}+\frac{1}{2h_1}(\mu_{k(\gamma)}-\boldsymbol{\nu}_{k(\gamma)})(\mu_{k(\gamma)}-\boldsymbol{\nu}_{k(\gamma)})^T|^{\frac{d_k+1}{2}}}$$

$$\mathbb{P}(\mu_{j(\gamma^c)} \mid \boldsymbol{\gamma}) = \left(\frac{1}{\sqrt{2\pi}}\right) \frac{\tilde{b}_j^{\tilde{a}_j}}{(\frac{\mu_{0j}^2}{2}+\tilde{b}_j)^{(\tilde{a}_j+\frac{1}{2})}} \frac{\Gamma(\tilde{a}_j+\frac{1}{2})}{\Gamma(\tilde{a}_j)},$$

where n_k is the number of subjects in the kth component.

We now provide more details on the sampling approach for the variable selection indicators $\boldsymbol{\gamma}$ and the component means $\boldsymbol{\mu}_{0k}$ that depend on these. To simplify the sampling for this probit prior, we follow the work of Albert and Chib (1993) and Chiang et al. (2017) and introduce a latent variable z_j, where

$$z_j \sim \mathcal{N}(\alpha_0 + \alpha_1 N_j, 1)$$
$$\gamma_j = \begin{cases} 1 & z_k > 0 \\ 0 & \text{otherwise.} \end{cases}$$

1.2.1 Sampling steps

Here we provide a summary of the MCMC algorithm, which, at a high level, consists of two steps. In each step, the given parameter under consideration is updated conditional on the current values of that parameter, as well as the additional parameters of interest:

1. The first step of the MCMC algorithm is a Metropolis-Hastings (MH) step on $\boldsymbol{\gamma}$, following the "add-delete-swap" approach to search over the space of possible feature combinations (Savitsky et al., 2011). Specifically, we choose an index j at random, and then propose changing the value of γ_j from 0 to 1 or from 1 to 0, or swapping two γ_j's with opposite values, accepting the proposed new $\boldsymbol{\gamma}^N$ over the original $\boldsymbol{\gamma}^O$ with probability:

$$\min\Bigg[1, \Bigg(\prod_{j:\gamma_j=0} \mathbb{P}(\mathbf{x}_{j(\gamma^c)} \mid \boldsymbol{\gamma}^N) \prod_{j:\gamma_j=1} \prod_{k=1}^{K} \mathbb{P}(\mathbf{x}_{jk(\gamma)} \mid \mu_{jk}, \boldsymbol{\gamma}^N) \prod_{k=1}^{K} \mathbb{P}(\boldsymbol{\mu}_{k(\gamma)} \mid \boldsymbol{\gamma}^N) \cdot \prod_{j:\gamma_j=0} \mathbb{P}(\mu_{0j} \mid \boldsymbol{\gamma}^N) \cdot \mathbb{P}(\boldsymbol{\gamma}^N) \Bigg) \Bigg/ \Bigg(\prod_{j:\gamma_j=0} \mathbb{P}(\mathbf{x}_{j(\gamma^c)} \mid \boldsymbol{\gamma}^O) \prod_{j:\gamma_j=1} \prod_{k=1}^{K} \mathbb{P}(\mathbf{x}_{jk(\gamma)} \mid \mu_{jk}, \boldsymbol{\gamma}^O) \cdot \prod_{k=1}^{K} \mathbb{P}(\boldsymbol{\mu}_{k(\gamma)} \mid \boldsymbol{\gamma}^O) \prod_{j:\gamma_j=0} \mathbb{P}(\mu_{0j} \mid \boldsymbol{\gamma}^O) \cdot \mathbb{P}(\boldsymbol{\gamma}^O) \Bigg) \Bigg] \tag{S3}$$

2. The next step is a random walk MH step to sample the $\boldsymbol{\mu}_{k(\gamma)}$. New values are proposed for every element μ_{kj} that is in the model at that iteration, from $\mu_{kj}^N = \mu_{kj}^O + \epsilon$, for $\epsilon \sim \mathcal{N}(0, v^2)$, for $j = 1, \ldots, p_\gamma$ and $k = 1, \ldots, K$. This transition is accepted with probability

$$\min\left[\frac{\mathbb{P}(\mathbf{x}_{jk(\gamma)} \mid \mu_{jk}^N, \boldsymbol{\gamma}) \mathbb{P}(\boldsymbol{\mu}_{k(\gamma)}^N \mid \boldsymbol{\gamma})}{\mathbb{P}(\mathbf{x}_{jk(\gamma)} \mid \mu_{jk}^O, \boldsymbol{\gamma}) \mathbb{P}(\boldsymbol{\mu}_{0k(\gamma)}^O \mid \boldsymbol{\gamma})}, 1 \right] \tag{S4}$$

1.2.2 Posterior summarization

Based on the sampled values for $\boldsymbol{\gamma}$, we can compute the marginal posterior probability of inclusion for each feature j as the MCMC average of γ_j. To identify a final set of selected features $\boldsymbol{\gamma}^*$, we consider the set of features with marginal posterior probabilities of inclusion above a certain threshold. Given the selected set of features, there are several possible approaches for obtaining posterior estimates of the corresponding $\boldsymbol{\mu}$ values. If there are sufficient MCMC iterations where the final set of selected features corresponds to the set of features included in that iteration, the posterior estimate of μ_{jk} for each selected feature j in each group k can be obtained as the average of the sampled values over those iterations. For settings with a larger number of selected predictors, this becomes a combinatorial search problem, and there may not be a sufficient number of iterations with the same configuration of $\boldsymbol{\gamma}$; instead, μ_{jk} can be

estimated as the marginal average for each selected predictor j over the iterations where it was included, or additional MCMC iterations can be run to resample $\boldsymbol{\mu}_{k(\gamma)}$ with $\boldsymbol{\gamma}$ fixed to the final set of selected features.

1.3 Prediction

The fitted model can also be used to make a classification prediction for newly observed data. If presented with a new observation with feature data $\mathbf{x}^{\text{new}}$, we use the predictive distribution of $\mathbf{x}^{\text{new}}$, $p_k(\mathbf{x}^{\text{new}}_{\boldsymbol{\gamma}^*})$, to classify the sample. Note that this distribution is using only the selected set of features $\boldsymbol{\gamma}^*$. For a given class k, this distribution is

$$p_k(\mathbf{x}^{\text{new}}_{\boldsymbol{\gamma}^*}) = \frac{1}{(2\pi)^{n_k/2}} \frac{(b'_k)^{(a'_k)}}{(b^{\text{new}}_k)^{(a^{\text{new}}_k)}} \frac{\Gamma(a^{\text{new}}_k)}{\Gamma(a'_k)}$$

where

$$a^{\text{new}}_k = a'_k + 1/2,$$
$$b^{\text{new}}_k = b_k + \frac{(\mathbf{x}_{k(\gamma^*)} - \mathbf{1}_{n_k}\mu_{jk})^T(\mathbf{x}_{k(\gamma^*)} - \mathbf{1}_{n_k}\mu_{jk})}{2}.$$

In b^{new}_k of this formulation, μ_{jk} is the posterior estimate computed as described in the previous section. We want to find the probabilities that the new data sample belongs to each of the groups k, given the existing observed data. Thus, using g^{new} as the class membership variable of the new observation, we want to find the prediction probability

$$\pi_k(g^{\text{new}} = k\,|\mathbf{X}) = p(g^{\text{new}} = k \mid \mathbf{x}^{\text{new}}, \mathbf{X}).$$

We assume that the membership proportion between classes is exchangeable between the training and future/validation data sets and so can estimate the probability of an observation being in a given group k, $\pi_k = \mathbb{P}(g = k)$, as $\hat{\pi} = \frac{n_k}{n}$. This assumption allows us to write the above equations together as

$$\pi_k(g^{\text{new}} = k \mid \mathbf{X}) = \frac{p_k(\mathbf{x}^{\text{new}}_{\boldsymbol{\gamma}^*})\hat{\pi}_k}{\sum_{i=1}^{K} p_i(\mathbf{x}^{\text{new}}_{\boldsymbol{\gamma}^*})\hat{\pi}_i}$$

The new data observation is then assigned to the class k for which this probability has the greatest value.

2 ADDITIONAL SIMULATION SCENARIOS

In this section, we summarize comparative performance for the following additional simulation scenarios: stronger class imbalance, varying number of predictors, varying signal strength, and varying ratio of discriminatory to non-discriminatory features. The basic simulation design is as in the primary simulation reported in Section 4.1.2 of the main manuscript. All simulation results reflect averages over 100 simulated data sets.

2.1 Stronger class imbalance

In addition to the even split (50% vs. 50%) and moderate imbalance (60% vs. 40%) settings summarized in Section 4 of the main manuscript, we performed an additional simulation study with a stronger class imbalance (90% vs. 10%), keeping the total sample size fixed at $n = 100$. Simulation results are summarized in terms of variable selection (Table S1) and classification accuracy on a test set (Table S2). The strong class imbalance makes both the variable selection and prediction tasks more challenging, resulting in lower MCC values for feature selection and lower Youden's Index for the prediction of class labels than in the simulations included in the main manuscript with either balanced or moderately imbalanced data.

	**TPR**	**FPR**	**MCC**
RVS	0.93	0.03	**0.75**
Neutral	0.74	0.03	0.68
Lasso	0.85	0.06	0.57

Table S1. Feature selection: The TPR, FPR, and MCC for feature selection when using data with an 90% vs. 10% class imbalance as described in Section 2.1.

	**TPR**	**FPR**	**Youden**
RVS	0.72	0.04	**0.68**
Neutral	0.66	0.05	0.57
Lasso	0.54	0.01	0.09
SVM	0.57	0.01	0.56

Table S2. Classification accuracy: The mean TPR, FPR, and Youden's index when using data with an 90% vs. 10% class imbalance as described in Section 2.1.

2.2 Varying number of predictors

The primary simulation setting includes 4 discriminatory features and 100 noise features. Here, we consider additional scenarios with varying p, keeping the ratio of discriminatory to noise features the same. Specifically, we considered scenarios with the following settings in terms of the number of discriminatory features p_d and the number of non-discriminatory features p_n: $p_d = 1$, $p_n = 25$; $p_d = 2$, $p_n = 50$; and $p_d = 8$, $p_n = 200$. Simulation results are summarized in terms of variable selection (Table S3) and classification accuracy on a test set (Table S4). In general, the results were consistent with the primary simulation, in that the proposed RVS method achieved the highest MCC for feature selection and highest Youden's index for classification. Across all methods, classification accuracy improved as the number of discriminatory predictors increased.

2.3 Varying signal strength

The primary simulation assumes a mean for the discriminatory features of 1 in the first group and -1 in the second group. Here, we consider reducing the signal strength by decreasing the separation between the group means for the discriminatory features. Specifically, we considered settings with group means for the discriminatory features of -0.6 in the first group vs. 0.6 in the second group, and -0.8 in the first group vs. 0.8 in the second group. The most noticeable trend was a decrease in Youden's index for correct group label prediction on the test data across all methods with decreasing separation between the groups.

	$p_d = 1, p_n = 25$			$p_d = 2, p_n = 50$			$p_d = 8, p_n = 200$		
	**TPR**	**FPR**	**MCC**	**TPR**	**FPR**	**MCC**	**TPR**	**FPR**	**MCC**
RVS	1.00	0.001	**0.99**	0.98	0.001	**0.98**	0.86	0.001	**0.91**
Neutral	0.81	0.001	0.96	0.69	0.002	0.82	0.42	0.001	0.61
Lasso	1.00	0.24	0.37	1.00	0.19	0.40	0.88	0.05	0.58

Table S3. Feature selection: The TPR, FPR, and MCC for feature selection with a varying number of discriminatory features (p_d) vs. non-discriminatory features (p_n), as described in Section 2.2.

	$p_d = 1, p_n = 25$			$p_d = 2, p_n = 50$			$p_d = 8, p_n = 200$		
	**TPR**	**FPR**	**Youden**	**TPR**	**FPR**	**Youden**	**TPR**	**FPR**	**Youden**
RVS	0.83	0.14	**0.69**	0.88	0.10	**0.77**	0.94	0.05	**0.90**
Neutral	0.83	0.16	0.56	0.86	0.14	0.68	0.91	0.09	0.82
Lasso	0.82	0.17	0.65	0.87	0.12	0.75	0.93	0.06	0.86
SVM	0.75	0.24	0.52	0.83	0.16	0.67	0.93	0.06	0.87

Table S4. Classification accuracy: The mean TPR, FPR, and Youden's index with a varying number of discriminatory features (p_d) vs. non-discriminatory features (p_n), as described in Section 2.2.

	Mean: -0.6 vs. 0.6			Mean: -0.8 vs. 0.8		
	**TPR**	**FPR**	**MCC**	**TPR**	**FPR**	**MCC**
RVS	0.86	0.0004	**0.91**	0.97	0.001	**0.98**
Neutral	0.67	0.0004	0.80	0.73	0.001	0.83
Lasso	0.96	0.09	0.54	0.99	0.10	0.52

Table S5. Feature selection: The TPR, FPR, and MCC for feature selection in simulation settings with varying signal strength, as described in Section 2.3.

	Mean: -0.6 vs. 0.6			Mean: -0.8 vs. 0.8		
	**TPR**	**FPR**	**Youden**	**TPR**	**FPR**	**Youden**
RVS	0.81	0.19	**0.63**	0.89	0.10	**0.78**
Neutral	0.79	0.21	0.56	0.87	0.13	0.74
Lasso	0.80	0.19	0.61	0.87	0.13	0.74
SVM	0.73	0.26	0.47	0.83	0.17	0.66

Table S6. Classification accuracy: The mean TPR, FPR, and Youden's index in simulation settings with varying signal strength, as described in Section 2.3.

2.4 Varying ratio of discriminatory to non-discriminatory features

In this section, we summarize simulation results for scenarios with the same number of non-discriminatory features as in the primary simulation ($p_n = 100$), but either fewer ($p_d = 2$) or more ($p = 10$) discriminatory variables. In the setting with very sparse signal, the lasso tended to select too many features, resulting in a higher FPR and lower MCC relatively to other simulation scenarios considered. Across all methods, the setting with a lower ratio of discriminatory features to noise features resulted in lower classification accuracy (Youden's Index) on the test set.

	**TPR**	**FPR**	**MCC**	**TPR**	**FPR**	**MCC**
RVS	0.96	0.001	**0.96**	0.84	0.001	**0.90**
Neutral	0.62	0.001	0.79	0.44	0.001	0.63
Lasso	1.00	0.12	0.38	0.79	0.07	0.60

Table S7. Feature selection: The TPR, FPR, and MCC for feature selection in simulation settings with varying ratio of discriminatory to non-discriminatory features, as described in Section 2.4.

	**TPR**	**FPR**	**Youden**	**TPR**	**FPR**	**Youden**
RVS	0.87	0.12	**0.75**	0.95	0.04	**0.91**
Neutral	0.84	0.14	0.63	0.92	0.07	0.85
Lasso	0.87	0.14	0.73	0.93	0.06	0.87
SVM	0.82	0.17	0.65	0.93	0.06	0.87

Table S8. Classification accuracy: The mean TPR, FPR, and Youden's index in simulation settings with varying ratio of discriminatory to non-discriminatory features, as described in Section 2.4.

2.5 Timing comparison

In this subsection, we consider the scalability of RVS, lasso, and SVM in terms of computing time for both increasing p and increasing n. All three methods were run using Matlab R2020b. We conducted simulations with various settings for p, the number of predictors, as described in Section 2.2. As shown in Figure S1, the proposed method (RVS) is slower than lasso or SVM; however, the time to run 100,000 MCMC iterations for $p = 208$ is still less than 1 minute on average, which is not exorbitant. As illustrated in Figure S2, we considered settings with n up to 10,000. These simulation results show that the timing for RVS was stable for increasing n, while the time for SVM increased relatively steeply for $n \geq 5000$.

3 SENSITIVITY ANALYSIS

In order to assess the model's sensitivity to changes in the setting of the hyperparameters, we noted how the posterior probability of inclusion (PPI) changes for both the discriminatory variables and the noise variables as the values of the hyperparameters were changed. The plots in Figure S3 show the change in the average PPI for discriminatory variables and noise variables in the simulation setting as the values for the three parameters c, α_0, and α_1 are varied.

4 CONVERGENCE

In this section, we provide additional details on the convergence of $\boldsymbol{\mu}$ in the RVS model for both the simulations and case study. Traceplots for the selected variables for an example simulated data set from the primary simulation described in Section 4.1.2 of the main manuscript are provided in Figure S4. Traceplots for the selected variables for the case study described in Section 4.2 of the main manuscript are provided in Figure S5. These plots were created using the `mcmc_trace()` function from the R package `bayesplot` (Gabry et al., 2019). $\hat{R}$ values were computed as described in Section 4.1.5 of the main manuscript. A histogram of the $\hat{R}$ values obtained across the 100 simulated data sets for the primary simulation described

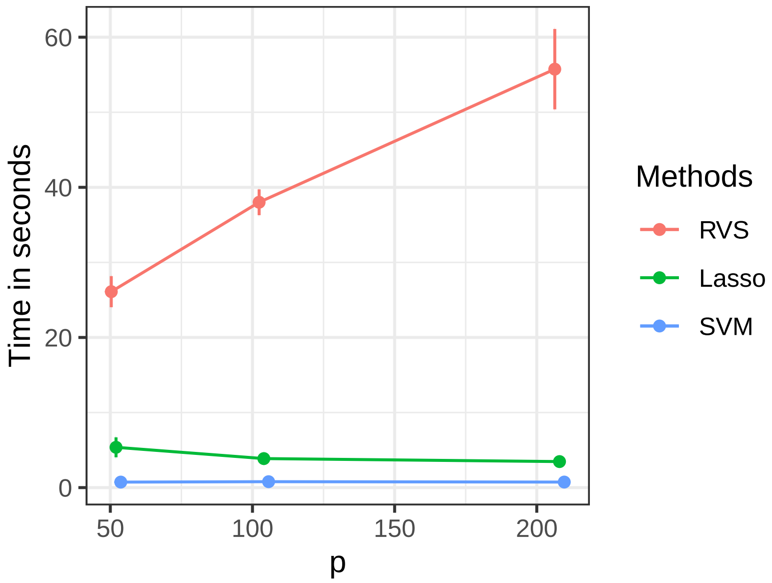

Figure S1: Timing comparison with increasing p. The x-axis shows the total number of predictors p, and the y-axis shows the mean computational time in seconds, along with a vertical bar for the 95% confidence interval across simulated data sets. For RVS, the runtime includes 100,000 MCMC iterations. For the lasso, the run time includes cross-validation for penalty parameter selection.

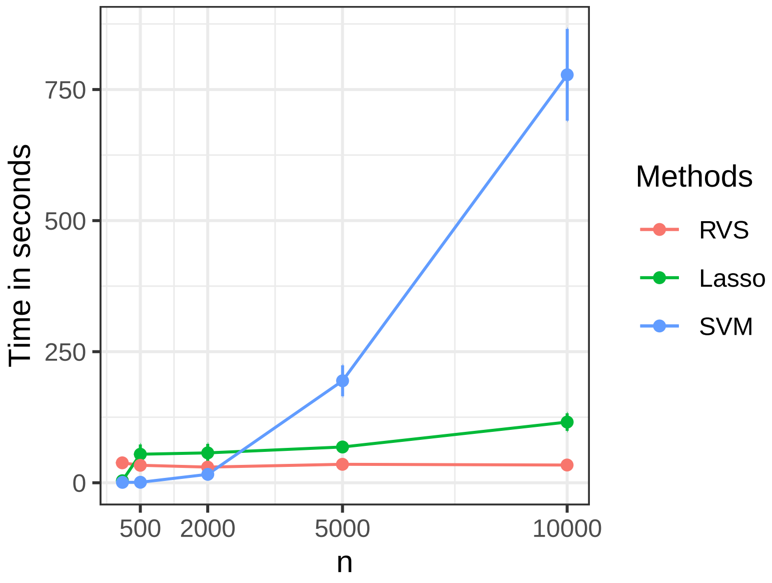

Figure S2: Timing comparison with increasing n. The x-axis shows the total number of samples n, and the y-axis shows the mean computational time in seconds, along with a vertical bar for the 95% confidence interval across simulated data sets. For RVS, the runtime includes 100,000 MCMC iterations. For the lasso, the run time includes cross-validation for penalty parameter selection.

in Section 4.1.2 of the main manuscript are provided in Figure S6. The mean $\hat{R}$ value was 1.002, the median was 1.001, and the maximum was 1.048. $\hat{R}$ values for the case study are reported in Table S9.

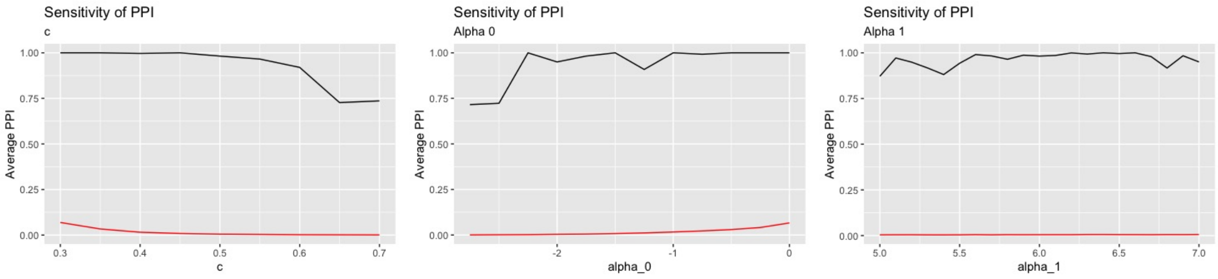

Figure S3: Change in average PPI for discriminatory variables (in black) and noise variables (in red) as the values for c, α_0, and α_1 are varied

We also explored how reducing the number of MCMC iterations affected convergence. When we ran shorter chains with 10,000 iterations as the basis for inference, the mean $\hat{R}$ value was 1.018, the median was 1.011, and the maximum was 1.374 across all simulated data sets. These results suggest that most chains converged well, but there were a limited number of cases with somewhat higher $\hat{R}$ values. Since the computational time was not prohibitive, we allowed for a more generous number of MCMC iterations to ensure convergence was reached for all features in all settings. For the case study, the mean, median, and maximum $\hat{R}$ values for $\boldsymbol{\mu}$ with 10,000 post-burn-in iterations were 1.0018, 1.0016, and 1.0044.

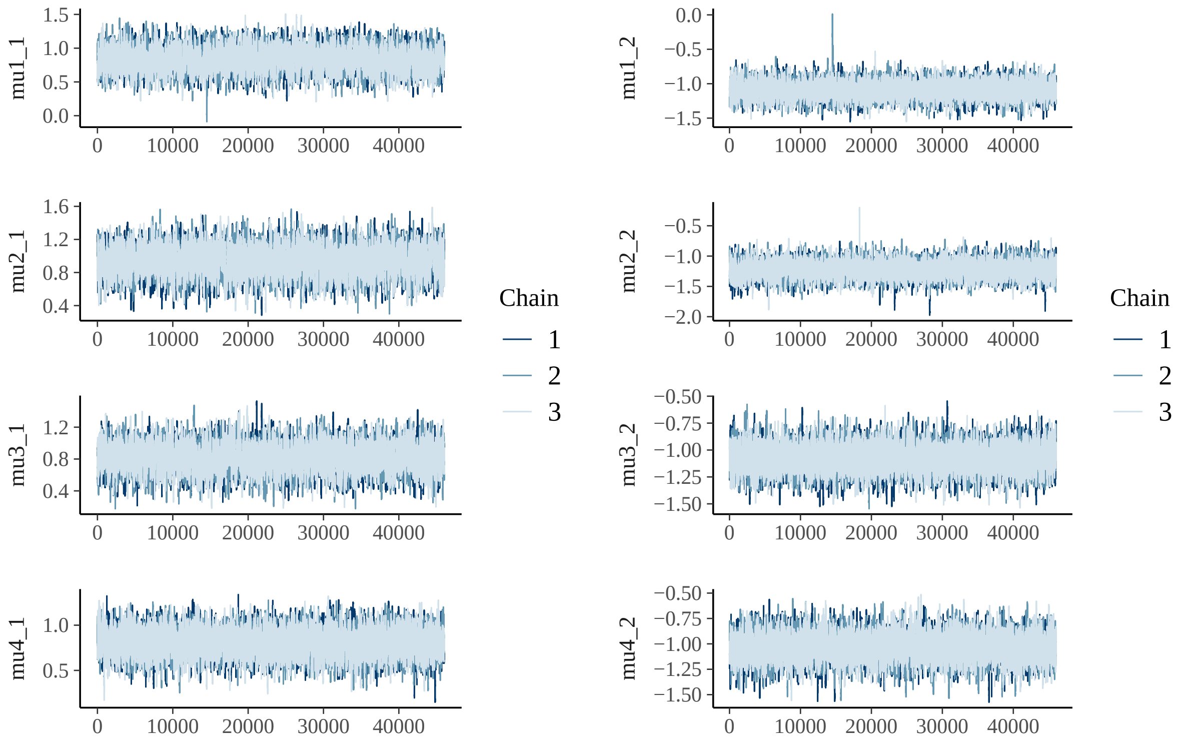

Figure S4: Traceplots for an example simulated data set

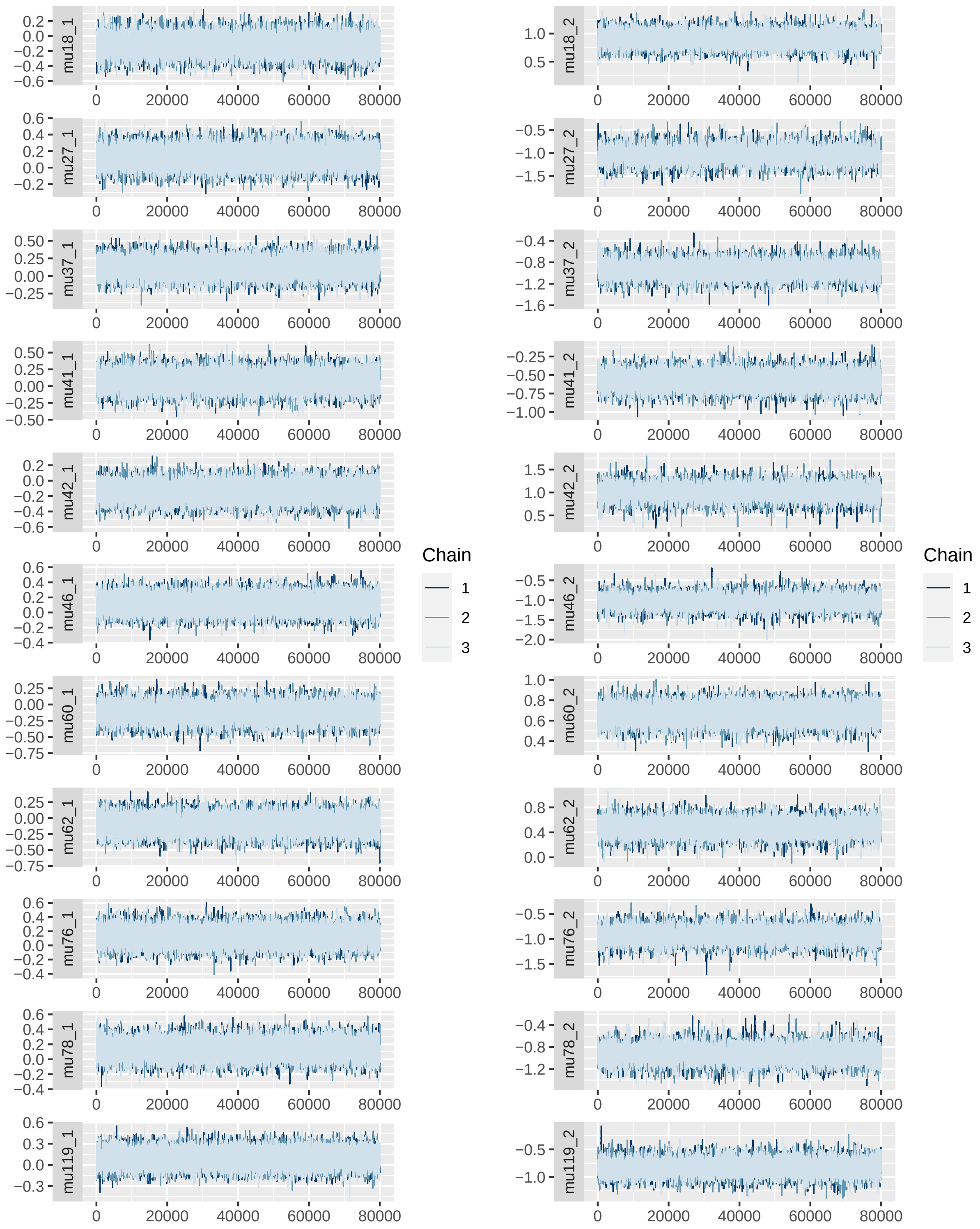

Figure S5: Traceplots for the case study

REFERENCES

Albert, J. H. and Chib, S. (1993). Bayesian analysis of binary and polychotomous response data. *Journal of the American Statistical Association* 88, 669–679

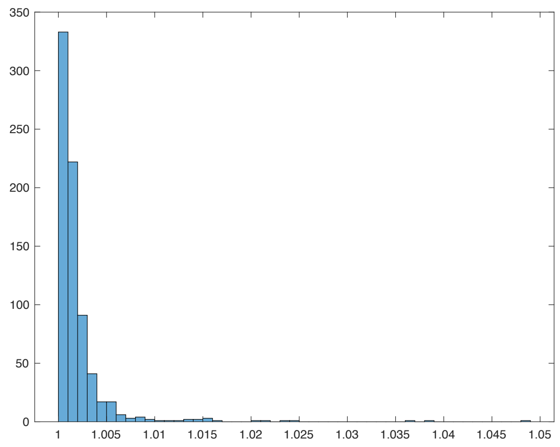

Figure S6: Histogram of $\hat{R}$ values for the primary simulation study

Class 1	Class 2
1.000247	1.000336
1.000343	1.000412
1.000500	1.000336
1.000098	1.000404
1.000737	1.001103
1.000217	1.000492
1.000363	1.000146
1.000319	1.000418
1.000474	1.000374
1.000126	1.001693
1.000256	1.000337

Table S9. $\hat{R}$ values for RVS on the case study. Each row corresponds to a selected variable, and the columns correspond to the classes

Carlin, B. P. and Chib, S. (1995). Bayesian model choice via Markov chain Monte Carlo methods. *Journal of the Royal Statistical Society: Series B (Methodological)* 57, 473–484

Chiang, S., Guindani, M., Yeh, H. J., Haneef, Z., Stern, J. M., and Vannucci, M. (2017). Bayesian vector autoregressive model for multi-subject effective connectivity inference using multi-modal neuroimaging data. *Human Brain Mapping* 38, 1311–1332

Dellaportas, P., Forster, J. J., and Ntzoufras, I. (2002). On Bayesian model and variable selection using MCMC. *Statistics and Computing* 12, 27–36

Gabry, J., Simpson, D., Vehtari, A., Betancourt, M., and Gelman, A. (2019). Visualization in bayesian workflow. *J. R. Stat. Soc. A* 182, 389–402. doi:10.1111/rssa.12378

Savitsky, T., Vannucci, M., and Sha, N. (2011). Variable selection for nonparametric gaussian process priors: Models and computational strategies. *Statistical Science* 26, 130
